# Modeling and measurement of lead tip heating and resonant length for implanted, insulated wires

**DOI:** 10.1002/mrm.30145

**Published:** 2024-05-31

**Authors:** Lydia J. Bardwell Speltz, Seung-Kyun Lee, Yunhong Shu, Matthew R. Tarasek, Joshua D. Trzasko, Thomas K. F. Foo, Matt A. Bernstein

**Affiliations:** 1 Department of Radiology, Mayo Clinic, Rochester, Minnesota, USA; 2 Mayo Clinic Graduate School of Biomedical Sciences, Mayo Clinic, Rochester, Minnesota, USA; 3 Technology and Innovation Center, GE HealthCare, GE Research, Niskayuna, New York, USA

**Keywords:** implanted devices, MR safety, resonant length, RF heating, wavenumber

## Abstract

**Purpose:**

To study implant lead tip heating because of the RF power deposition by developing mathematical models and comparing them with measurements acquired at 1.5 T and 3 T, especially to predict resonant length.

**Theory and Methods:**

A simple exponential model and an adapted transmission line model for the electric field transfer function were developed. A set of wavenumbers, including that calculated from insulated antenna theory (King wavenumber) and that of the embedding medium were considered. Experiments on insulated, capped wires of varying lengths were performed to determine maximum temperature rise under RF exposure. The results are compared with model predictions from analytical expressions derived under the assumption of a constant electric field, and with those numerically calculated from spatially varying, simulated electric fields from body coil transmission. Simple expressions for the resonant length bounded between one-quarter and one-half wavelength are developed based on the roots of transcendental equations.

**Results:**

The King wavenumber for both models more closely matched the experimental data with a maximum root mean square error of 9.81°C at 1.5 T and 5.71°C at 3 T compared to other wavenumbers with a maximum root mean square error of 27.52°C at 1.5 T and 22.01°C for 3 T. Resonant length was more accurately predicted compared to values solely based on the embedding medium.

**Conclusion:**

Analytical expressions were developed for implanted lead heating and resonant lengths under specific assumptions. The value of the wavenumber has a strong effect on the model predictions. Our work could be used to better manage implanted device lead tip heating.

## 1 | INTRODUCTION

A major safety concern for patients undergoing an MRI exam with active implanted devices or abandoned leads is device heating, especially at lead tips, due to RF power deposition.^1–3^ Because of this, devices often have labeled conditions that restrict certain kinds of scans. For example, although 3 T MRI has become the standard of care for brain exams,^4^ device labeling often precludes its use for patients with some implanted devices. Further modeling to estimate lead tip heating and resonant length conditions could aid device design and labeling. Resonant length corresponds to maximal RF heating, and its accurate prediction is important to avoid thermal injury.^5^ It can correspond to half the RF wavelength,^5–7^ under conditions we will explore in this article.

A standard method to estimate lead tip heating is to calculate the lead tip voltage with the complex line integral of the tangential component of the incident electric field along the implanted lead,^8^ with a suitable multiplicative transfer function $h\left(l\right)$. Park et al.^2^ used this method to calculate lead tip heating from a straight wire in a uniform electric field. Other transfer functions have been proposed; however, some^9,10^ did not explicitly account for resonant length or current loss effects that can be modeled with the complex wavenumber, k (i.e., spatial frequency expressed in units of rad m^−1^). Other earlier studies^3,6,11^ adopted the complex wavenumber of the embedding medium to calculate the resonant length. More recently, Liu et al.^12^ introduced a transmission line model to calculate induced voltage at the device (proximal) end of the lead, and Wang et al.^13^ experimentally investigated dependence of abandoned lead tip heating on proximal end termination without focus on theoretical modeling. Additionally, a modified transmission line model was introduced by Acikel et al.^14^ to determine induced current in a device lead, with the effect on temperature rise at 3 T completed in a later study.^15^

In this work, we report modeling and measurements of lead tip temperature rise at 1.5 T and 3 T for a set of straight, insulated wires of varying lengths in a phantom. The measurements provide insight into the dependence of temperature rise on the lead length, with the maximum temperature rise occurring at the resonant length. We compare the measurements with the line integral calculations based on different transfer function models and values of the complex wavenumber, k. The calculation is performed for both a constant electric field, as considered in Park et al.^2^ and Yeung et al.,^3^ and also for a spatially dependent electric field simulated from a whole-body RF coil, as done in Alon et al.^9^ and Golestanirad et al.^10^ In the constant electric field case, we develop analytical expressions for the temperature rise (up to a single free parameter for overall scaling) as a function of the lead length, d, as well as expressions for the resonant length derived with no free parameters.

The value of the wavenumber is expected to have a strong effect on the transfer function and the resonant length. We use the wavenumber calculated for an insulated conductor, as determined from the antenna theory by King et al.^16^ Previously, this wavenumber, which we call the King wavenumber, was used in transmission line models^12^ and to model electrically short implants.^17^ We compare the results from using the King wavenumber versus the wavenumbers of the embedding medium (e.g., phantom gel, water, or tissue), which have previously been used to estimate the resonant length, as reported in texts on MR safety, (e.g., McRobbie).^5^

For the functional form of the transfer function, we first consider a simple exponential model,^18^ which yields closed-form expressions in the constant electric field case, and includes, as a special case, the choice of a unity transfer function $h\left(l\right)=1$, which has been used in some analyses, (e.g., Golestanirad et al.^10^) To overcome limitations of the simple exponential model, we then compare it to a more sophisticated transmission line model of Liu et al.^12^ The model was originally introduced to estimate induced voltage at the implantable pulse generator (IPG) end of a lead, but can be adapted to calculate lead tip voltage, as done here.

## 2 | THEORY

Biological tissue can be characterized electromagnetically by its permittivity, ϵ in F/m, permeability, μ in H/m, and conductivity, σ in S/m. According to standard electromagnetic theory,^19^ the complex wavenumber, $k_{t}$, in a medium such as tissue is given by:

(1) Rekt=ωϵμ21+σϵω2+112,Imkt=ωϵμ21+σϵω2−112,

where ω/2π is the Larmor frequency measured in Hz, and μ is permeability. As is typical for MR applications, we take μ to be the permeability of free space, $\mu_{0}=4\pi \times 10^{-7}$ H/m.

The complex wavenumber k inside an insulated conducting wire was derived in King et al.^16^ as a function of the wavenumber $k_{t}$ of the embedding medium and the wavenumber $k_{i}$ of the insulator, which are both calculated from Equation (1):

(2) $$k=k_{i}{\left(1+\frac{F\left(k_{t}b\right)}{\ln\left(b/a\right)}\right)}^{1/2},\;F\left(z\right)=\frac{H_{0}^{\left(1\right)}\left(z\right)}{zH_{1}^{\left(1\right)}\left(z\right)}.$$

**FIGURE 1:**
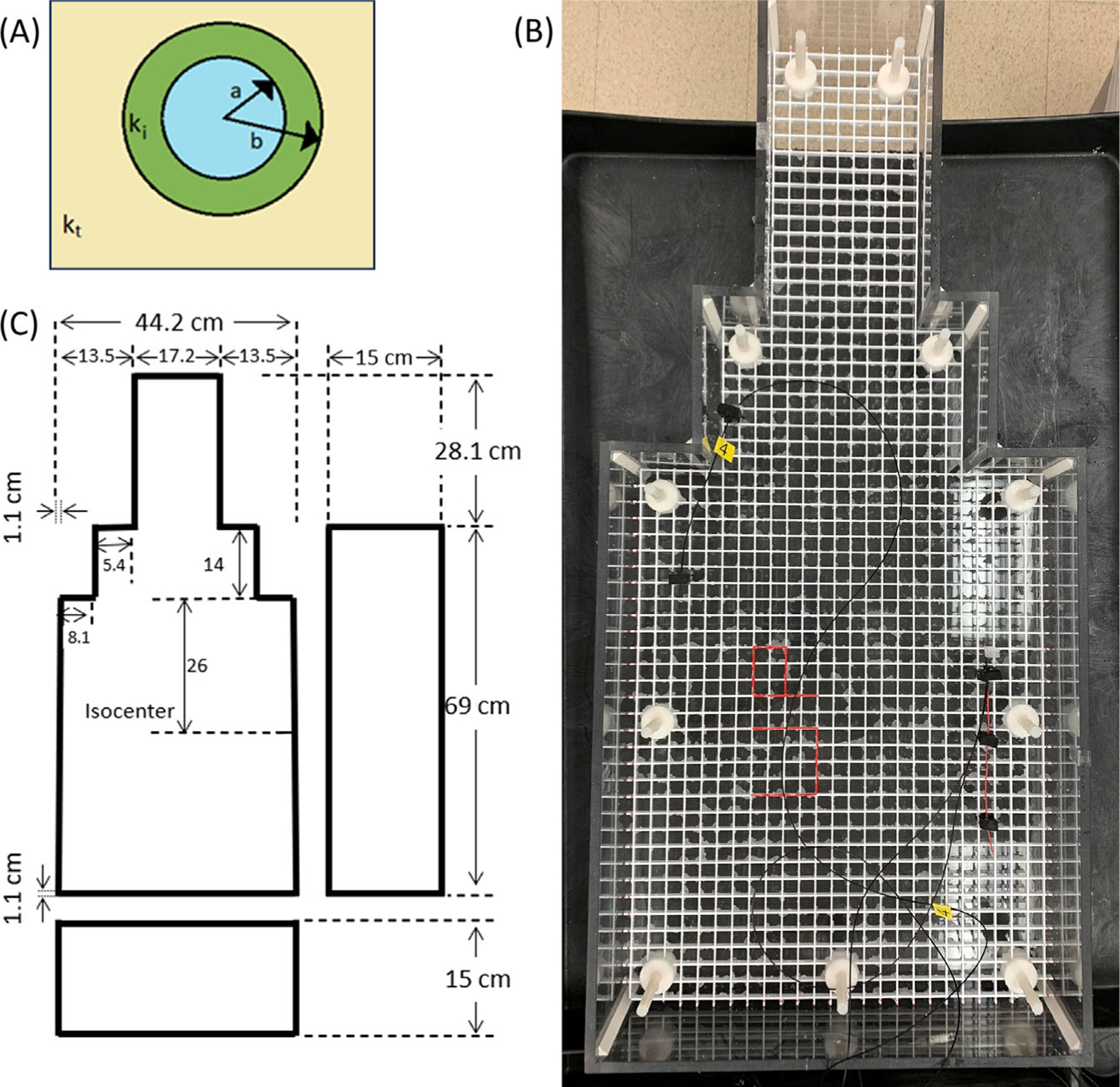
(A) Cross-section of central conducting wire, insulating layer i, embedded in tissue t. The wavenumber k in the conductor is calculated from insulated antenna theory as described in King et al.^16^ (B) ASTM phantom with a PVC grid submerged to help support the wires as well as temperature probes. The set of 15 wires had lengths of (in cm): 2.38, 10.38, 16.38, 20.38, 21.38, 22.38, 26.38. 29.38, 32.78, 35.78, 39.78, 40.78, 41.78, 25.78, and 50.78. The implantable pulse generator, (IPG) end of each wire was covered with a silicone gel to replicate a wire capped at one end (i.e., the plastic end case described in Wang et al.^13^). The “lead tip” end of each wire had 5 mm of the insulation removed. The wires were placed along the right side of the phantom (patient left) as shown, where the electric field is strongest so that a robust temperature rise could be measured. (C). Physical dimensions of the ASTM phantom. ASTM, American Society for Testing and Materials.

Here, $H_{0}^{\left(1\right)}\left(z\right)$ and $H_{1}^{\left(1\right)}\left(z\right)$ are Hankel functions of the first kind of order 0 and 1, respectively,^20^ and a and b are the inner and outer radii of the insulating layer as shown in Figure 1A. Because the insulating layer is assumed to have σ=0, its wavenumber $k_{i}$ is real and reduces to $k_{i}=\omega \sqrt{\epsilon \mu }$. In Equation (2), all other quantities can be complex, except for the radii a and b. This King wavenumber is valid for a conductive embedding material,^16^ so as described in Liu et al.,^12^ is less accurate when the embedding tissue is mostly composed of fat.

Lead tip voltage can be calculated^2,12^ from the line integral along the entire length of the lead of the tangential component $E_{t}$ of the incident electric field vector E multiplied by a transfer function $h\left(l\right)$:

(3) $$V=\int_{0}^{d}h\left(l\right)\text{E}\left(l\right)\cdot dl,$$

where l is measured from the lead tip, d is the total length of the lead, and bold font indicates a vector. In general, both the transfer function $h\left(l\right)$ and the incident electric field vector can be complex quantities.

The tangential component of the electric field $E_{t}$ is frequently used in calculations of the line integral.^2,21^ Given a short segment of wire whose path is described by the real vector Δl, the tangential component of the electric field can be calculated with the scalar product:

(4) $$E_{t}\left(l\right)=\frac{\text{E}\left(l\right)\cdot \text{Δ}l}{\left\|\text{Δ}l\right\|}.$$

Note that because the vector electric field E can be a complex quantity, $E_{t}$ can be as well.

The predicted lead tip temperature rise ΔT is then calculated,^2,13^ up to an unknown scaling constant, from the absolute square of the voltage:

(5) $$\text{Δ}T\propto {\left|V\right|}^{2}.$$

Equation (5) reflects Ohmic losses from current flowing from the lead tip, or equivalently the well-known quadratic dependence of specific absorption rate on electric field.^5^

### 2.1 | Simple exponential model for the transfer function

In the simple exponential model,^18^ the transfer function is taken to be:

(6) $$h\left(l\right)=e^{-ikl},$$

where $k=k_{R}+ik_{I}$ is the complex King wavenumber of the conductor given by Equation (2) with $k_{R}$ and $k_{I}$ being its real and imaginary components, respectively. The motivation behind the simple exponential model is to account for resonant length and loss effects with the simplest possible transfer function. Because Equations (1) and (2) contain one-half powers (i.e., square roots) there is latitude for the sign choice of $k_{R}$ and $k_{I}$, which will become important when we calculate resonant length. For reasons that will become clearer later, we chose (−) sign in the exponent of Equation (6) to be consistent with the sign convention of Liu et al.^12^ Consequently, we make the corresponding sign choice for the square root in Equation (2) to ensure that $k_{I}\leq 0$. Physically, this means that the imaginary part of the wavenumber produces losses, rather than unbounded gains in the transfer function. The real part of the wavenumber $k_{R}$ is related to the wavelength λ in the conductor by:

(7) $$k_{R}=\frac{2\pi }{\lambda }.$$

As such, we will assume that $k_{R}\geq 0$. A further comment on the sign of *k*_*I*_ is provided in Discussion.

First, we consider a special case where the electric field vector component along the path of the wire is a complex constant denoted by E, for example, this could be the z-component of the vector E. Equation (3) can be evaluated analytically and yields:

(8) $$V=\frac{E}{-ik}\left(\left(e^{k_{I}d}\cos\left(k_{R}d\right)-1\right)-ie^{k_{I}d}\sin\left(k_{R}d\right)\right).$$

From Equations (5–8), we expect the lead tip temperature rise to be

(9) $$\text{Δ}T\propto \frac{{\left|E\right|}^{2}}{{\left|k\right|}^{2}}\left(1+e^{2k_{I}d}-2e^{k_{I}d}\cos k_{R}d\right),$$

where ${\left|k\right|}^{2}=k_{R}^{2}+k_{I}^{2}$. The resonant length can then be determined by taking the derivative of Equation (9) with respect to d, setting it equal to zero, and solving for the smallest, non-zero positive root of the transcendental equation. Assuming ${\left|k\right|}^{2}>0$ and removing a common factor of $2e^{k_{I}d}{\left|E\right|}^{2}/{\left|k\right|}^{2}$ yields:

(10) $$\left(k_{I}e^{k_{I}d}-k_{I}\cos k_{R}d+k_{R}\sin k_{R}d\right)=0.$$

We now introduce two non-negative, dimensionless variables $u=-k_{I}/k_{R}$ and $g=k_{R}d/\text{π}$. From Equation (7), g is the length d measured in units of half-wavelengths. Then Equation (10) can be re-expressed as:

(11) $$-ue^{-\pi ug}+u\cos\left(\pi g\right)+\sin\left(\pi g\right)=0.$$

Note that if $k_{I}=0$, that is, the conductor is not lossy because of radiative effects, then u=0, and from Equation (11) g=1, that is, the resonant length $d_{\text{res,sem}}$ is one-half wavelength for the simple exponential model:

(12) $$k_{R}d_{\text{res},\text{sem}}=\pi \text{ or }d_{\text{res},\text{sem}}=\lambda /2.$$

Similarly, from Equation (11) as u→∞, $\cos\left(\pi g\right)$ must approach zero, so the resonant wavelength approaches λ/4. For other values, we can consider $g\left(u\right)$ to be an implicitly defined function of u, and the resonant length solution for the simple exponential model is

(13) $$d_{\text{res},\text{sem}}=\frac{\lambda }{2}\times g_{\text{sem}}\left(u\right).$$

We can solve the transcendental Equation (11) numerically, and some values of $g_{\text{sem}}\left(u\right)$ are provided in Table 1. The resulting values for the resonant length are bounded by λ/2 (when u=0), and λ/4 (as u→∞). Values of the resonant length for values of u not provided in Table 1 can be obtained with the publicly available MATLAB-based calculator^22^ associated with this article.

We also can expand Equation (9) for the short wire case $\left(\left|k\right|d\ll 1\right)$ to demonstrate that the simple exponential transfer function predicts quadratic dependence of temperature rise on lead length:

(14) $$\begin{array}{l} \text{Δ}T\propto \frac{{\left|E\right|}^{2}}{{\left|k\right|}^{2}}\left(k_{R}^{2}d^{2}+k_{I}^{2}d^{2}\right)+\ldots ={\left|E\right|}^{2}d^{2}+\ldots ,\\ \left|k\right|d\ll 1,\;\left(\mathrm{short}\;\mathrm{wire}\;\mathrm{case}\right). \end{array}$$

### 2.2 | Transmission line model for the transfer function

Liu et al.^12^ described a transmission line model for the lead using the principle of reciprocity. As described in Liu et al.,^12^ the model was originally applied to calculate the voltage at the IPG end of the lead, but it can readily be adapted to calculate the lead tip voltage using symmetry. Following Equations (6–9) in Liu et al.^12^ when the impedance of the “IPG” (i.e., the capped end) is much greater than the impedance of the lead, the transfer function reduces to:

(15) $$h\left(l^{\prime }\right)\propto \frac{e^{-ik\left(d-l^{\prime }\right)}\left(1-\Gamma e^{-2ikl^{\prime }}\right)}{\left(1-\Gamma e^{-2ikd}\right)},$$

where Γ is the reflection coefficient at the capped end and $l^{\prime }$ is measured from the IPG end. To obtain a transfer function analogous to Equation (6) that satisfies the boundary condition $h\left(0\right)=1$, we adapt Equation (15) by making the substitution $l=d-l^{\prime }$, yielding:

(16) $$h\left(l\right)\propto \frac{e^{-ikl}\left(1-\Gamma e^{-2ik\left(d-l\right)}\right)}{\left(1-\Gamma e^{-2ikd}\right)},$$

Although Equation (16) reduces to the simple exponential model when the reflection coefficient Γ=0, Equation (15) does not.

Assuming a constant electric field, substituting Equations (16) into (3) yields an expression for the complex voltage at the lead tip.


(17) $$V=\frac{E}{ik}\left(\frac{1}{e^{ikd}-\Gamma e^{-ikd}}\right)\left(\left(e^{ikd}-1\right)+\Gamma \left(e^{-ikd}-1\right)\right),$$

In the following analysis, we will assume that Γ is real. The case of a complex reflection coefficient can be considered by retaining its imaginary part in Equation (17), or alternatively evaluating the line integral in Equation (3) numerically. Analogous to Equation (9), we can derive a closed form expression for lead tip temperature rise under the condition of a constant electric field:

(18) $$\begin{array}{l} \text{Δ}T\propto \frac{{\left|E\right|}^{2}}{{\left|k\right|}^{2}}\times \frac{1}{e^{-2k_{I}d}+\Gamma^{2}e^{+2k_{I}d}-2\Gamma \cos2k_{R}d}\\ \times \left[\left(e^{-2k_{I}d}+\Gamma^{2}e^{2k_{I}d}\right)+2\Gamma \cos2k_{R}d+{\left(1+\Gamma \right)}^{2}-2\left(1+\Gamma \right)\cos k_{R}d\left(e^{-k_{I}d}+\Gamma e^{+k_{I}d}\right)\right]. \end{array}$$

The resonant length for the transmission line model can be found by the same procedure to derive Equations (10) and (11), but because of the complexity of the expressions it might be simpler to numerically maximize Equation (18), instead of constructing an analytical solution. An exception occurs for the physically interesting case of Γ=1, corresponding to an electrical open circuit (see Discussion), under which Equation (18) reduces to manageable trigonometric and hyperbolic functions. Using the identities ${\left|\cos\left(z\right)\right|}^{2}=\cos^{2}\left(x\right)+\sinh^{2}y$ and ${\left|\sin\left(z\right)\right|}^{2}=\sin^{2}\left(x\right)+\sinh^{2}y$, with z=x+iy, we can apply Equation (5), take the derivative of ΔT with respect to length d, and set it equal to zero. After some algebra and applying further identities, we find the following condition for the resonant length

(19) $$\left({\left(\cos\left(k_{R}d\right)-\cosh\left(k_{I}d\right)\right)}^{2}\times (k_{I}\cos\left(k_{R}d\right)\sinh\left(k_{I}d\right)+k_{R}\sin\left(k_{R}d\right)\cosh\left(k_{I}d\right)\right)=0.$$

The first factor in Equation (19) can never be zero when $\left|k\right|>0$, so we conclude that the resonant length is equal to the smallest, positive solution to the relatively simple transcendental equation:

(20) $$k_{R}\tan\left(k_{R}d\right)+k_{I}\tanh\left(k_{I}d\right)=0.$$

Solving for the resonant length in the transmission line model with Γ=1 yields

(21) $$d_{\text{res},\Gamma 1}=\frac{\lambda }{2}\times g_{\Gamma 1}\left(u\right),$$

where $g_{\Gamma 1}$ is tabulated in Table 1.

Like its counterpart from the simple exponential model Equation (13), the resonant length for the transmission line model is bounded by λ/2 and λ/4 when Γ=1. This can be readily seen from Table 1, or directly from Equation (20) because both $k_{R}$ and the product $k_{I}\tanh\left(k_{I}d\right)$ are never negative, implying that the argument of the tangent function $k_{R}d$ for the smallest positive root of Equation (19) must lie in the second quadrant, that is, between $\frac{\pi }{2}$ and π.

For the short wire case $\left(\left|k\right|d\ll 1\right)$, Equations (17) or (18) can be expanded analogously to Equation (14). At least two limiting cases emerge, both of which, like Equation (14), display a quadratic dependence of temperature rise on length in the short wire case:

(22) $$\text{Δ}T\propto \left\{\begin{array}{ll} {\left|E\right|}^{2}d^{2}+\ldots , & \left|k\right|d\ll \frac{\left|\Gamma -1\right|}{\left|\Gamma +1\right|}\\ \frac{{\left|E\right|}^{2}d^{2}}{4}+\ldots , & \frac{\left|\Gamma -1\right|}{\left|\Gamma +1\right|}\ll \left|k\right|d\ll 1 \end{array}\right..$$

The temperature rise in the first line of Equation (22) for short wire is the same as the prediction of the simple exponential model Equation (14). Interestingly, the second line of Equation (22) predicts that the temperature rise is reduced by a factor of four for an equivalent electric field for a short wire when Γ≈1.

Finally, recall that all the theoretical results in this section were derived under the assumption of a constant electric field. We note that from Equation (3), because a constant electric field is symmetric, that is, $\text{E}\left(l\right)=\text{E}\left(d-l\right)$, Equations (17–22) remain unchanged regardless of whether the expression in Equations (15) or (16) is used for the transfer function. For the case of a spatially varying electric field vector, the analytical results derived in this section are no longer directly applicable, but are replaced by evaluating Equation (3) numerically with discrete summation and a transfer function from Equations (6) or (16). Additionally, it is worth noting that given an arbitrary electric field vector E obtained from simulations, we can obtain its tangential component $E_{t}$ using Equation (4) if the path of the wire is known. In practice, the wire path can be determined from an X-ray or CT data, as described in Golestanirad et al.^10^ and Bardwell et al.^18^

## 3 | METHODS

### 3.1 | ASTM phantom

An ASTM phantom was used to host the straight wires of varying lengths, one at a time. The phantom was built according to the ASTM F2182–11a^23^ for implant heating. The gelled saline had a conductivity of 0.47 S/m and a relative permittivity of $\varepsilon /\varepsilon_{0}=80$.

### 3.2 | Temperature measurements

**FIGURE 2:**
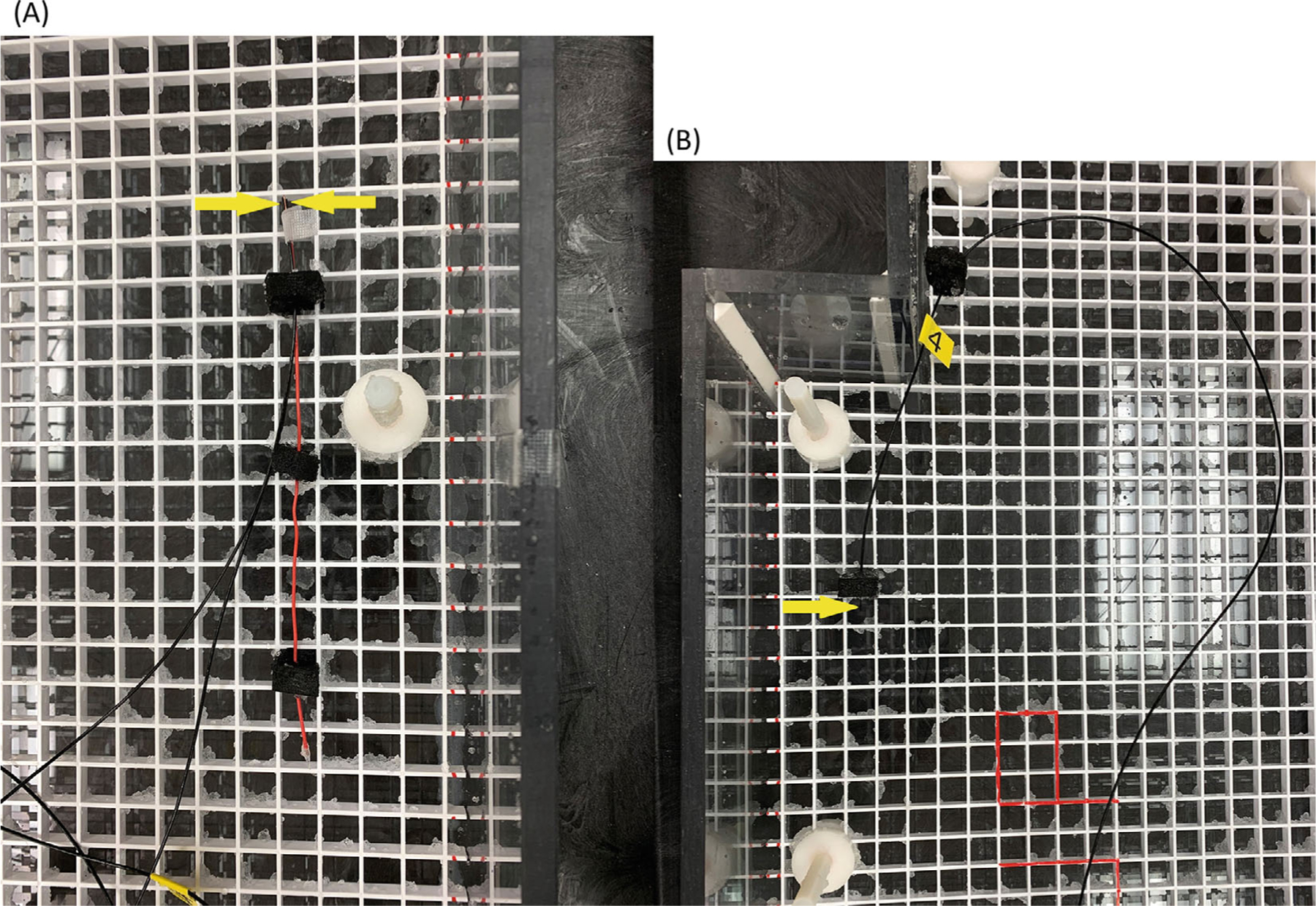
ASTM phantom with the locations (yellow arrows) of two fluoroptic probes at the wire tip (A) and the reference probe (B). ASTM, American Society for Testing and Materials.

**FIGURE 3:**
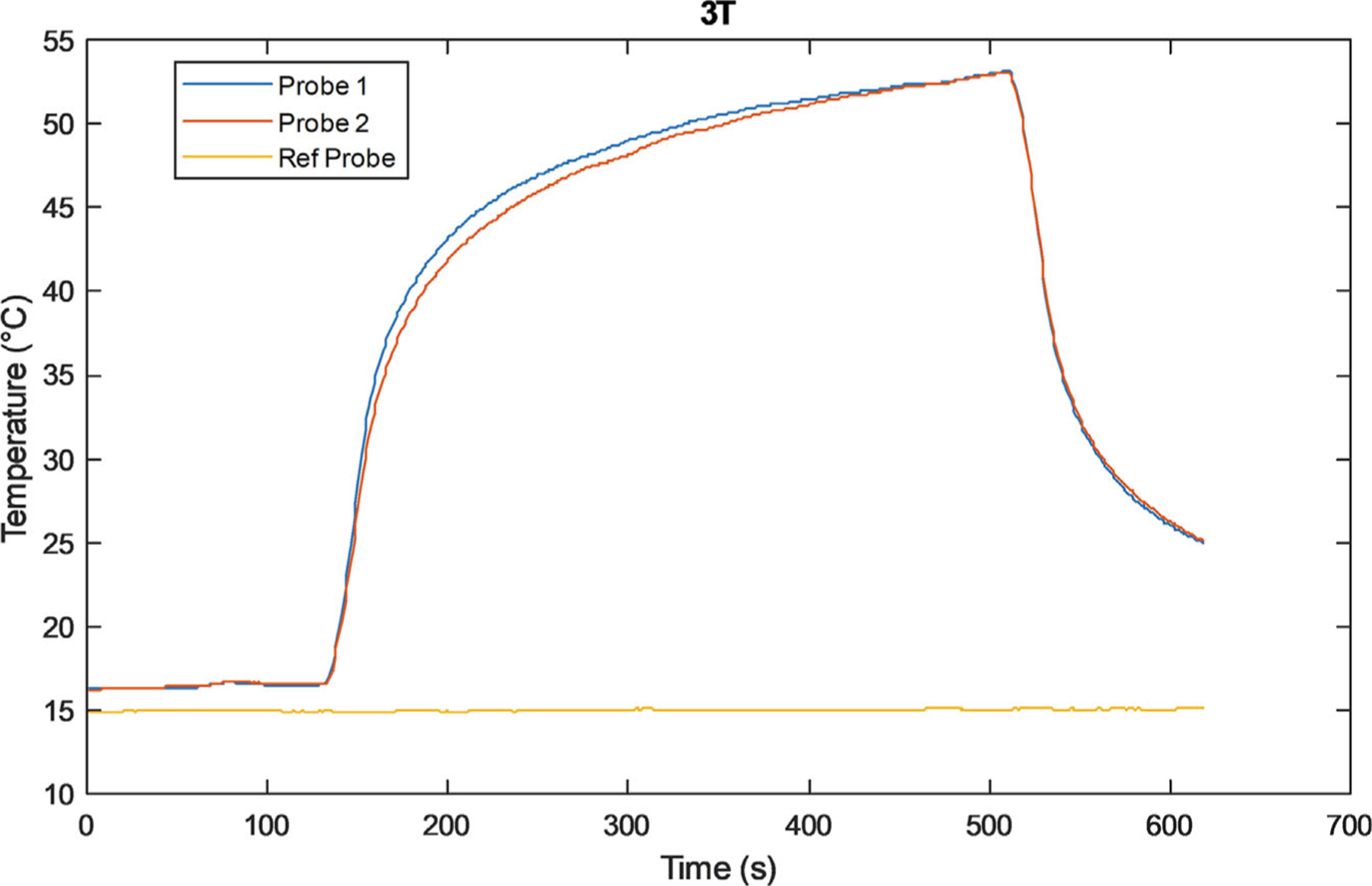
Representative temperature versus time data at 3 T for the 29 cm-long wire. All measured lead tip temperature rises were obtained by processing the acquired temperature versus time plots with a 120 s baseline, 360 s of RF exposure, and a 120 s cooling off period. Temperature data was smoothed using a 10 s moving average in MATLAB (The MathWorks) with the two probes located by the wire tip being averaged. The maximum change in temperature was calculated by the difference between the last 10 s of RF exposure and the last 10 s of the baseline.

Temperature measurements at wire tips were taken on 1.5 T and 3 T scanners (GE HealthCare) using an RF body coil for transmission. The set of 15 wires with lengths (in cm) varying from 2.38 to 50.78 was placed as illustrated in Figure 1B. Temperature measurements were performed using a Fluoroptic thermometer (Luxtron model FOT Lab Kit, Lumasense Technologies) with a resolution of 0.1°C. The sampling rate was 10 s^−1^. For each exam, table position was set so isocenter corresponded to middle of the “torso” (see Figure 1C) and the center of each wire was placed at isocenter to create the most uniform electric field achievable over the wire, with the aim of testing Equations (9) and (18). Three temperature probes (model STF-2) were used, two at less than 1 mm from the lead tip and one as a reference away from the tip, as shown in Figure 2. All temperature measurements were taken while scanning with an axial T_2_-weighted fast spin-echo (FSE) pulse sequence. A representative temperature versus time plot is shown in Figure 3.

The FSE sequence has a nominal RF output to achieve the prescribed flip angles, resulting in an associated whole-body specific absorption rate (SAR) calculated by the scanner software (given in Table S1). Auto prescan is run to adjust the RF transmit gain (TG) (measure in units of 1/10 of decibels, i.e., 30 counts corresponding to a factor of two in RF power) to calibrate RF power so that nominal RF output is achieved. Because of their differences in length, each wire j can load the RF coil slightly differently, yielding different auto prescan TG values (TG_APS*j*_) for some wires.

In our experimental design, after preliminary testing we manually selected a single, applied TG (TG_s_) at each field strength to produce an approximate 50°C maximum temperature rise at the lead tip for the expected resonant length. That TG_s_ was applied for all wire lengths at a given field strength. The effective SARs at those TG values are given in Table S1.

To account for the slight variation in TG_APS_ among the wires at each field strength, four runs of auto prescan were completed to obtain an average TG_APS*j*_ for each wire j. The corrected temperature rise $\text{Δ}T_{c}$ was calculated by:

(23) $$\text{Δ}T_{c}\left(j\right)=\text{Δ}T_{\text{measured}}\left(j\right)\times 10^{\left({\text{TG}}_{\text{APSj}}-{\text{TG}}_{\text{APSref}}\right)/100}.$$

The TG-corrected values $\text{Δ}T_{c}\left(j\right)$ in Equation (23) are what we would expect to obtain if the sequences for all the wires had the same flip angles as a reference wire, at the applied transmit gain TG_s_. Overall effect of this TG correction was relatively minor, as shown in Figure S1.

### 3.3 | Constant electric field comparison for different transfer functions and wavenumbers

The TG-corrected experimental data showing temperature rise was compared with model predictions developed in Theory for the simple exponential model of Equation (9) and the transmission line model of Equation (18). Note that these models only predict temperature rise as a function of wire length up to a single, unknown scaling factor.

To calculate the King wavenumber for the wires, we set $\epsilon =2.3\epsilon_{0}$ based on the data sheet for the insulated wire we used,^24^ and radii a=0.390 and b=0.625 mm. For reference, we also generated the results of the simple exponential model Equation (9) using wavenumber of the ASTM gel and the wavenumber of water, as calculated with Equation (1), and the results for the unity transfer function $h\left(l\right)=1$, corresponding to k=0.

The measured data were also compared with the transmission line model Equation (18) using the King wavenumber, as described in Methods. Note that unlike the simple exponential model, which only has a single free parameter (i.e., an overall scaling), the transmission line model generally has two free parameters, the overall scale factor and the reflection coefficient, Γ. We considered two cases related to Γ. First, we allowed Γ to vary as a free parameter and selected the value that resulted in the smallest root mean square error (RMSE) for a given field strength. Second, we set Γ=1, which has the physical motivation of complete reflection because the end opposite from the lead tip was capped with an insulator and has high impedance, that is, electrically open. Note, with the choice of Γ=1 in the transmission line model there is only one free parameter (i.e., an overall scale factor) like the simple exponential model.

For all models, the resonant length was calculated, without any free parameters.

### 3.4 | Simulated, spatially varying electric field comparison for different transfer functions and wavenumbers

**FIGURE 4:**
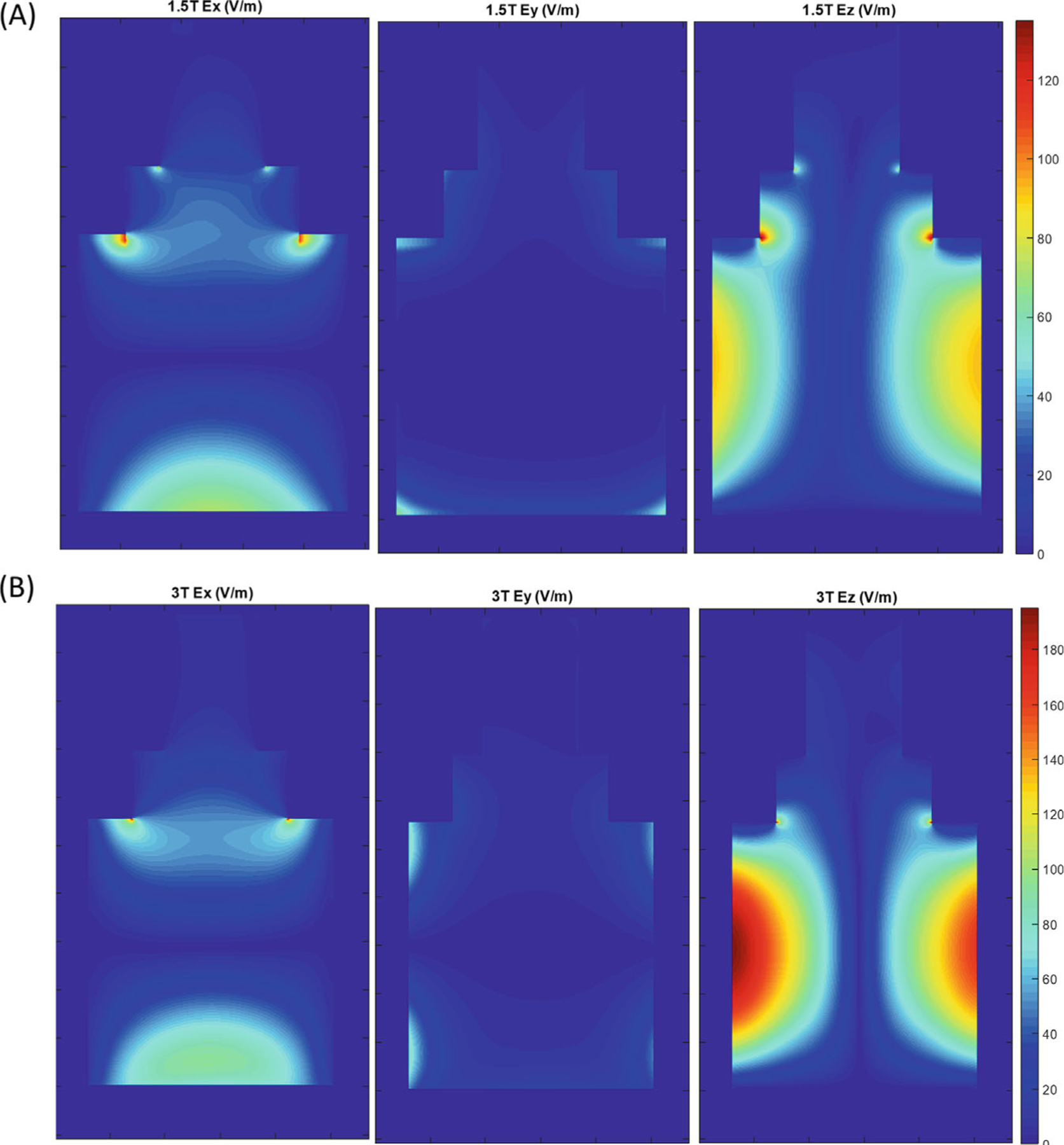
Electric field simulations for the ASTM phantom at *y* = 0 (i.e., a coronal midline slice) at 1.5 T (A) and at 3 T (B). ASTM, American Society for Testing and Materials.

The calculated temperature rise predictions as a function of lead length were repeated using a spatially varying, simulated electric field and numerical integration of Equation (3) via discrete summation. The electric fields of a 3 T and 1.5 T body transmit coil were simulated with the ASTM phantom using Sim4Life (ZMT) as described in Bardwell et al.^18^ and Tarasek et al.^25^ These simulations yield a spatial map of the complex electric field E, from which we can calculate the tangential component using Equation (4). The simulation placed the RF isocenter at the location matched to the experimental landmark for 1.5 T and 3 T (Figure 4). As seen in Figure 4, the strongest E-field occurs along the *z*-direction, with the highest values laterally along the left or right hand side of the phantom. Although wires rarely follow a perfectly straight path in clinical placement, this configuration for our experiments was chosen to obtain a robust temperature increase. Examining the simulated E-field in the phantom, we can see that along many paths $\left|E_{z}\right|\gg \left|E_{x}\right|$ and $\left|E_{z}\right|\gg \left|E_{y}\right|$, in which case, the line integral is dominated by the $E_{z}$ term.

### 3.5 | Statistics

**FIGURE 5:**
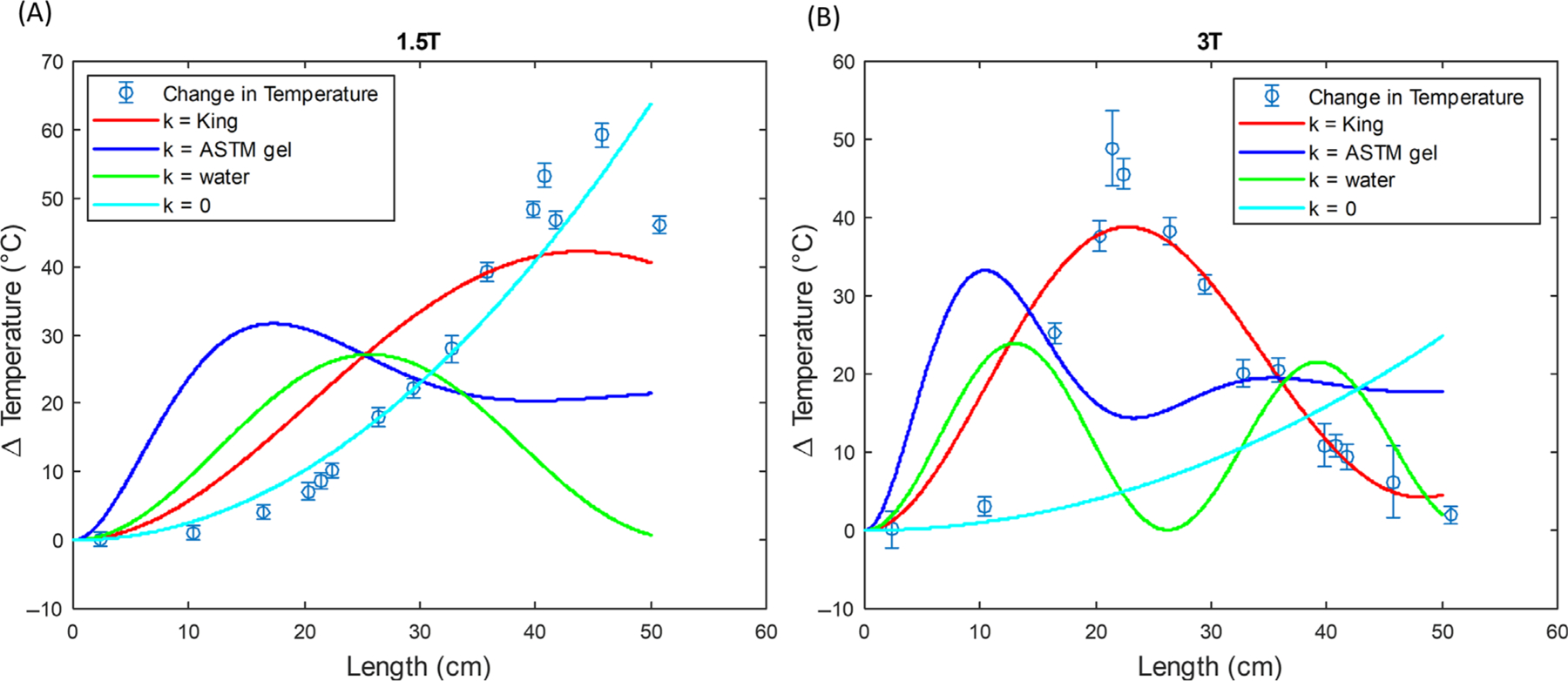
Predicted temperature rise ΔT for simple exponential model (SEM) considering different values for the wavenumber and a constant electric field at 1.5 T (A) and 3 T (B).

**FIGURE 6:**
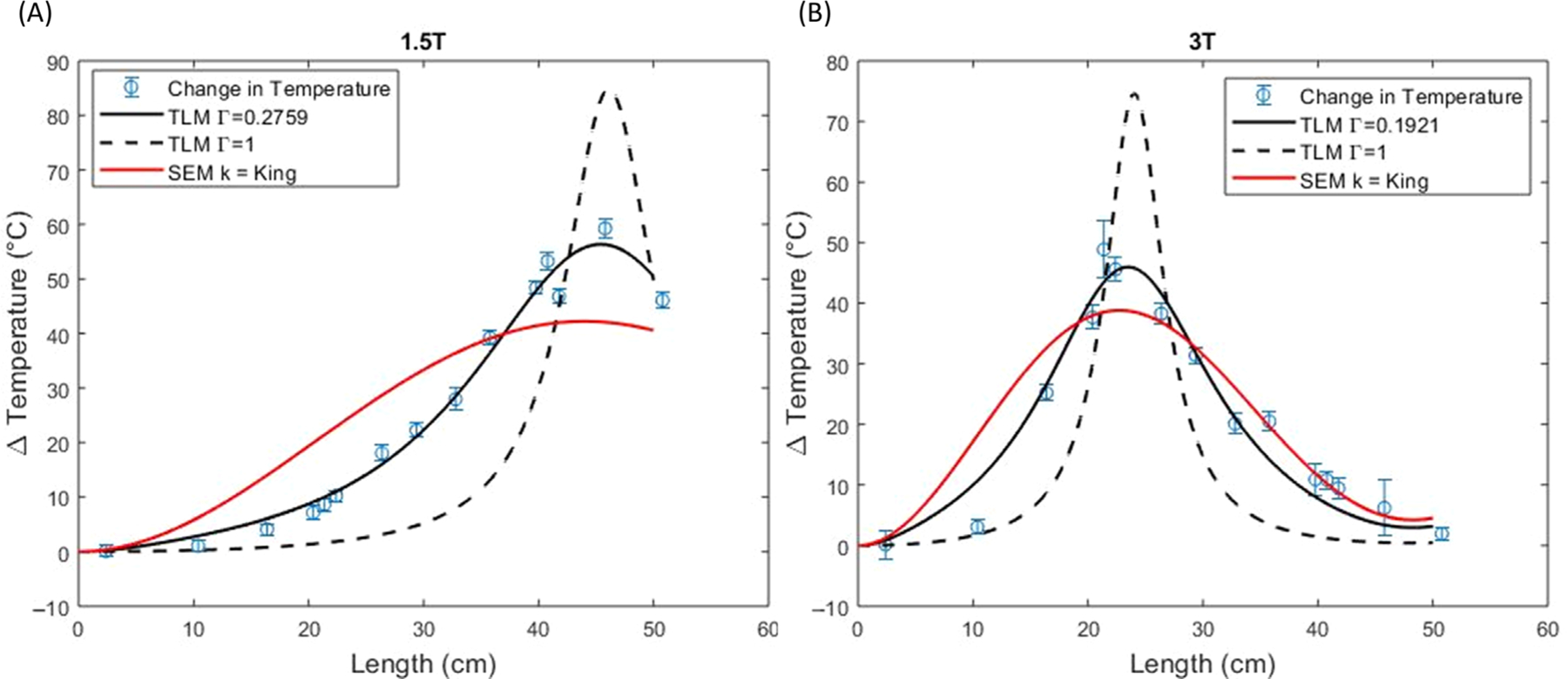
Predicted temperature rise ΔT from the transmission line model (TLM) using an optimized Γ or fixed value of Γ=1, compared to the simple exponential model (SEM) using the King wavenumber from Figure 5 for a constant electric field at 1.5 T (A) and 3 T (B).

**FIGURE 7:**
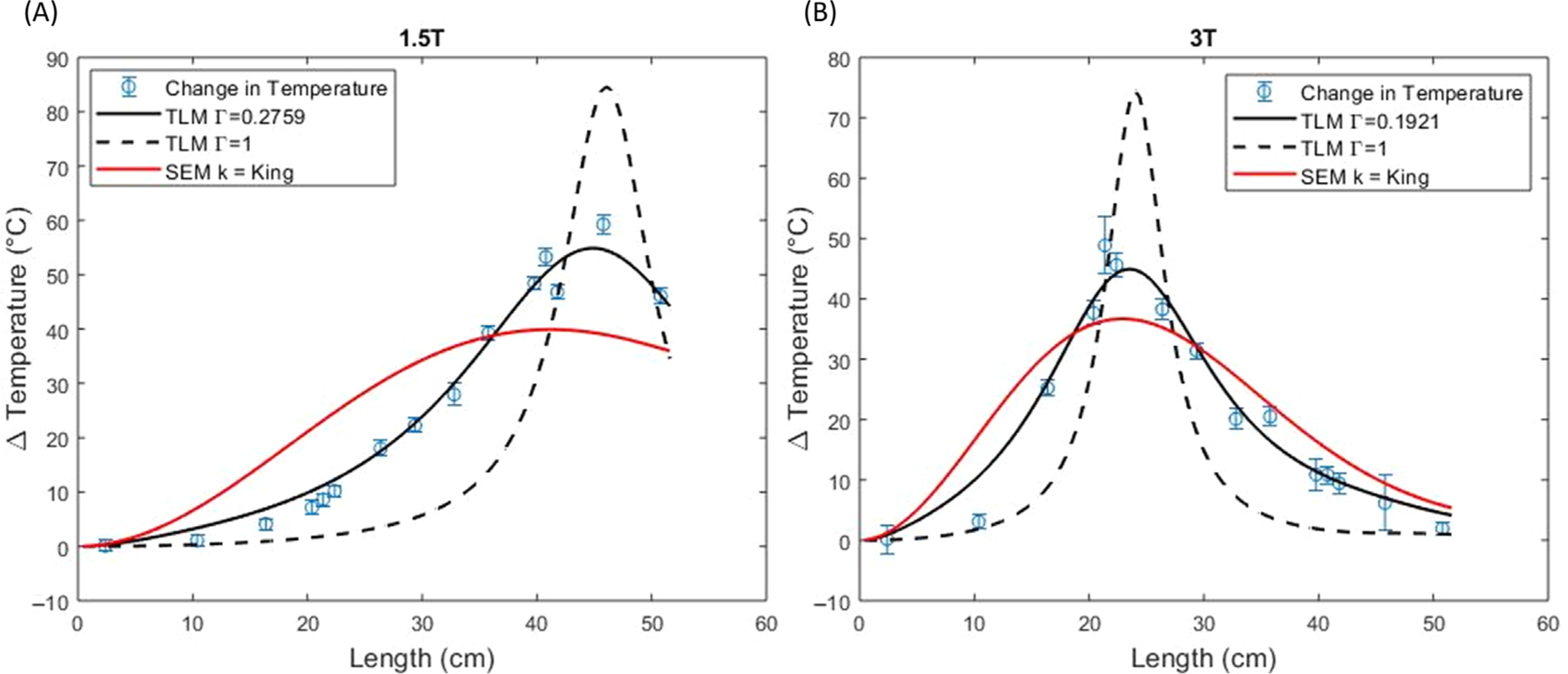
Simple exponential model (SEM) using the King wavenumber compared to the transmission line model (TLM) using an optimized Γ or fixed value of Γ=1 for a simulated electric field at 1.5 T (A) and 3 T (B). In this case, Equation (3) is evaluated numerically.

We accounted for three sources of error for the temperature measurements. The first source of error is the difference in temperature rise measured by the two different fluoroptic probes placed at the lead tip. The second arises from any variation in TG values that were measured during the four different auto prescan runs for each wire described by Equation (23). The final source of error is the SD of the baseline temperature measurement before any RF power was applied. The square root of the sum of the squares of these three source errors yielded the error bars shown in Figures 5–7.

An independent scale factor for each of the model predictions was fit to the temperature data to minimize the RMSE. The simple exponential model had one overall scaling factor that was optimized to obtain the smallest RMSE. The transmission line model contained two free parameters unless Γ=1, as described early. Because we do not have absolute truth data, the Akaike information criterion (AIC) was calculated to compare these different models because they have a different number of free parameters. This was calculated using the ordinary least squares as shown in Banks and Joyner.^26^

### 3.6 | Publicly available tools

To promote reproducible research, a MATLAB-based calculator for the wavenumber, the lead length dependence of lead-tip voltage and temperature rise (up to an undetermined scale factor), and the resonant length (assuming constant electric field) with the various transfer function models that we describe in this article was created. The data that support the findings of this study are openly available in GitHub at (https://github.com/ljbardwell/Resonant-Length-Predictor).^22^ The user can input several parameters to calculate the relevant embedding medium and King wavenumbers, the resonant lengths, and the lead length dependence of predicted lead tip voltage and temperature rise.

## 4 | RESULTS

### 4.1 | Wavenumber calculations at 1.5 T and 3 T

The wavenumber of the medium (i.e., ASTM gel) is (25.6650–9.2351*i*) rad m^−1^ at 3 T, and (14.4974–8.1746*i*) rad m^−1^ at 1.5 T, as calculated from Equation (1) (see Discussion for a note about the sign convention for $k_{I}$ pertaining to Equation (2)). The wavenumber of the insulator is 4.0602 rad m^−1^ at 3 T and 2.0301 rad m^−1^ at 1.5 T calculated from Equation (1), assuming σ=0. Finally, the King wavenumber is calculated using the wavenumbers of the insulator and medium using Equation (2), and is equal to (12.8679–1.6601*i*) rad m^−1^ at 3 T and (6.7454–0.6840*i*) rad m^−1^ at 1.5 T.

### 4.2 | Constant electric field comparison for different transfer functions and wavenumbers

Figure 5 shows the fitted ΔT predictions for the simple exponential model using the King wavenumber, the wavenumber of the ASTM gel, the wavenumber of water, and the unity transfer function compared to the experimental data at 1.5 T and 3 T. Figure 6 shows the simple exponential model using the King wavenumber compared to the transmission line model (Equation (18)). Two values of Γ are shown: a fixed Γ=1 and an optimized Γ that is allowed to freely vary to minimize the RMSE. The optimized values of Γ are 0.2759 at 1.5 T and 0.1921 at 3 T.

### 4.3 | Simulated, spatially varying electric field comparison for different transfer functions and wavenumbers

Figure 7 shows the simple exponential model using the King wavenumber compared to the two models using the transmission line transfer function shown in Equation (16), one with an optimized Γ and the other with a Γ=1. The same optimized Γ value was used as in the constant electric field case; Γ=0.2759 at 1.5 T and Γ=0.1921 at 3 T.

### 4.4 | Metrics to assess the various models

Table 2 lists the resonant length, the number of free parameters for the ΔT fit, the RMSE, and the AIC, for the simple exponential models and transmission line models at 1.5 T and 3 T assuming a constant electric field, as well as the simulated electric field.

For all the results provided in Table 2 (i.e., both field strengths and both constant and simulated electric fields) the transmission line model with optimized Γ yielded the best fits with the lowest RMSE and AIC.

As also can be seen from Table 2, at both 1.5 and 3 T, the choice of wavenumber has a large effect on the fits and the resonant lengths. The King wavenumber for both models more closely matched the experimental data with a maximum RMSE of 9.81°C at 1.5 T and 5.71°C at 3 T compared to other wavenumbers such as ASTM gel and water with a maximum RMSE of 27.52°C at 1.5 T and 22.01°C for 3 T. The resonant length was also more accurately predicted and differed substantially from values solely based on the embedding medium.

## 5 | DISCUSSION

An accurate prediction of the resonant length is important to estimate lead tip heating conditions. In some MR safety texts, 26 and 13 cm are sometimes quoted as the resonant lengths for 1.5 T and 3 T, respectively.^5^ However, as described in Theory, and as seen from the data in Figure 5 and Table 2, the complex wavenumber has a strong effect on the predicted resonant length. Our measurements suggest resonant lengths of ∼45 and 21 cm at 1.5 T and 3 T, respectively, for these wires. Both the simple exponential model and transmission line models using King wavenumber predict similar resonant lengths that are in good agreement with our measurements, without the use of any free parameters. Note that this difference in resonant lengths is not a new result and has been reported previously.^2,3,27,28^ Here we provide theoretical development and a publicly available calculator that can be used to predict such resonant length based on given input parameters.^22^

There are many other parameters that can vary in practice and will affect resonant length, including the permittivity of the insulation, the insulation thickness, and the properties of the embedding tissue. For example, Figure S2 shows the predicted resonant length and associated parameters using the transmission line model with an optimized Γ for insulation thickness ranging from our experimental value to a factor of three thicker. As the insulation thickness increases, the model predicts a steady increase in resonant length. This behavior is consistent with another study examining the effect of increased insulation.^3^

The fits for ΔT shown in Figure 5 and Table 2 are useful to assess the models developed in Theory. We think the 3 T data are more informative because, unlike 1.5 T, they include wire lengths d that are considerably longer than the resonant length. For example, the simple exponential model with the unity transfer function fits very well at 1.5 T, but fails poorly at 3 T because it cannot account for resonant length. That is, when $h\left(l\right)=1$ and the electric field is constant, Equations (3) and (5) have the simple solution $\text{Δ}T\propto {\left|E\right|}^{2}d^{2}$, which never rolls over for large d. Considering the 1.5 T and 3 T data in aggregate suggests the importance of using the King wavenumber to predict the resonant length and temperature rise.

The transmission line model was previously tested experimentally for different types of lead termination on the IPG side (i.e., metal-end vs. plastic-end).^13^ The new experiments reported here correspond to the end opposite from the lead tip being insulated, that is, the plastic-end case. Within this model, we considered two cases applying Equation (18) to fit the data: setting Γ=1, and allowing Γ to be optimized as a free parameter, which resulted in the best fit in terms of lowest AIC (i.e., the least information lost). Despite this curve fitting result, perhaps the choice of Γ=1 is also physically reasonable, because it is consistent with capping the lead with an insulator, provides a good prediction of resonant length, and a reasonably good fit to ΔT, with only a single free parameter for overall scaling. However, from Table 2, use of the King wavenumber in the simple exponential model, which also has only a single free parameter, outperformed the transmission line model with Γ=1. This result is consistent with the fitted values of the reflection coefficient Γ being closer to zero than to one.

There are several limitations to this study. The measurements are restricted to straight, insulated wires that were not actual device leads, and their ends were capped with insulators. Although this may be a reasonable model for capped abandoned leads, it does not capture many clinical situations, including leads connected to IPGs, leads capped with non-insulators,^13^ and helical leads.^17^ Nevertheless, by assuming the impedance at IPG end was much greater than the device lead, we were able to develop analytical expressions within the transmission line model, which provided considerable insight. To further develop these models, additional phantom experiments will be required. Additionally, this work did not explore the effect wire loops have on heating, which can be substantial. For example, Vu et al.^29^ reported the effect on RF heating when changing the size and placement of deep brain stimulation leads with loops. In future work, we plan to add the effect of wire loops to the models reported here.

Another limitation of the study is that we did not independently optimize Γ for the simulated electric field case because of computational complexity. However, because the transmission line model with the simulated electric field in Figure 7 appears similar to the transmission line model with the constant electric field in Figure 6, we think adopting Γ from the constant electric field is a reasonable assumption.

We mentioned earlier that the signs of the real and imaginary part of the wavenumber are open to choice because of the square roots of Equations (1) and (2). Although the real part $k_{R}$ is never negative because of Equation (7), Liu et al.^12^ and King et al.^16^ adopted opposite sign conventions for $k_{I}$. Here, we used the convention $k_{I}\leq 0$ in agreement with Liu et al.^12^ for all equations in this article, except for $k_{t}$ only when evaluating Equation (2), because King adopted the sign convention $k_{I}\geq 0$ (page 1695 in King et al.^16^).

After these models have been further validated, they could provide useful guidance in clinical settings as well as for device companies. Clinically, they could guide lead placement, as well as how to avoid resonant lengths if a lead needs to be cut and abandoned. The MATLAB-based tools^22^ could promote the wider use of the King wavelength and provide a better estimate of resonant length. For example, device manufacturers have some latitude in choosing the radii of the leads and insulating layers, as well as the dielectric constant of the insulating layer, which directly affect the wavenumber, which in turn affects resonant length.

## 6 | CONCLUSIONS

This study reported phantom measurements of maximum lead tip temperature rise at both 1.5 T and 3 T for a set of insulated straight wires of varying lengths. The results were compared with model predictions based on several candidate transfer functions and values of the complex wavenumber. Overall, a simple exponential model and a transmission line model provided good predictions for resonant length and the dependence of lead tip temperature rise on lead length, with the latter model yielding the lowest AIC. Without any free parameters, we derived expressions for the resonant length assuming constant electric field, and showed cases where the resonant wavelength must be bounded between one-quarter and one-half the wavelength in the conductor. Overall, our results also strongly suggest the use of a wavenumber derived from insulated antenna theory, the King wavenumber, which provides more accurate modeling compared to other wavenumbers, including that of the embedding medium, for both 1.5 T and 3 T.

## Supplementary Material

Fig S1

Tab S1

Fig S2

## Figures and Tables

**TABLE 1:** Tabulated values of the function $g\left(u\right)$ used to calculate the resonant length.

u	$g_{\text{sem}}\left(u\right)$	$g_{\Gamma 1}\left(u\right)$
0	1	1
0.05	0.970445	0.997526
0.1	0.944714	0.990407
0.15	0.921981	0.979433
0.2	0.901668	0.965641
0.25	0.883350	0.950053
0.3	0.866706	0.933533
0.35	0.851487	0.916733
0.4	0.837496	0.900104
0.45	0.824574	0.883939
0.5	0.812591	0.868413
0.6	0.791026	0.839597
0.7	0.772124	0.813860
0.8	0.755394	0.791024
0.9	0.740465	0.770782
1.0	0.727052	0.752809
5	0.562788	0.562833
∞	0.5	0.5

*Note*: Tabulated values of the function $g\left(u\right)$ used to calculate the resonant length with Equations (12) or (21) under the simple exponential model or transmission line model with Γ=1, respectively, and $u=-k_{I}/k_{R}$. Consistent with those equations, $g\left(0\right)=1$, and it smoothly decreases to its asymptotic value of $g\left(u\right)\to \frac{1}{2}$ as u→∞.

**TABLE 2:** Statistics and resonant length prediction at 1.5 T and 3 T for the SEM and TLM.

	1.5 T				3 T			
Model	Resonant length (cm)	No. of free parameters	RMSE (°C)	AIC	Resonant length (cm)	No. of free parameters	RMSE (°C)	AIC
**Constant electric field**								
SEM k = King	43.95	1	9.81	17.94	22.75	1	5.71	9.82
SEM k = ASTM gel	17.30	1	22.77	30.57	10.40	1	17.96	27.01
SEM k = water	25.65	1	27.52	33.42	12.95	1	22.01	30.06
SEM k = 0	n/a	1	6.78	12.41	n/a	1	22.79	30.59
TLM optimized Γ	45.40	2	2.36	−1.43	23.50	2	3.63	5.03
TLM Γ=1	46.10	1	14.39	23.68	24.05	1	10.11	18.40
**Simulated electric field**								
SEM k = King	41.20	1	11.43	20.24	22.80	1	6.51	11.78
TLM optimized Γ	44.80	2	2.59	−0.01	23.60	2	3.09	2.62
TLM Γ=1	46.00	1	13.95	23.22	24.00	1	9.90	18.08

*Note*: The predicted resonant length, number of free parameters for the ΔT fit, RMSE, AIC for relevant models at both 1.5 T and 3 T, considering both a constant electric field and a simulated electric field.

Abbreviations: AIC, Akaike information criterion; ASTM, American Society for Testing and Materials; RMSE, root mean square error; SEM, simple exponential model; TLM, transmission line model.

## Data Availability

An interactive MATLAB-based calculator for the wavenumber, the lead length dependence of lead-tip voltage and temperature rise (up to an undetermined scale factor), and the resonant length (assuming constant electric field) with the various transfer function models described in this article is available at https://github.com/ljbardwell/Resonant-Length-Predictor (commit: dabd2da). The user can input several parameters to calculate the relevant embedding medium and King wavenumbers, the resonant lengths, and the lead length dependence of predicted lead tip voltage and temperature rise.
