# Supplementary material for: Modeling and measurement of lead tip heating and resonant length for implanted, insulated wires
Source: Magn Reson Med. Author manuscript; Available in PMC 2025 Oct 1. (PMC11414523; doi:10.1002/mrm.30145)
Supplement: Fig S1 [file NIHMS1990163-supplement-Fig_S1.docx]

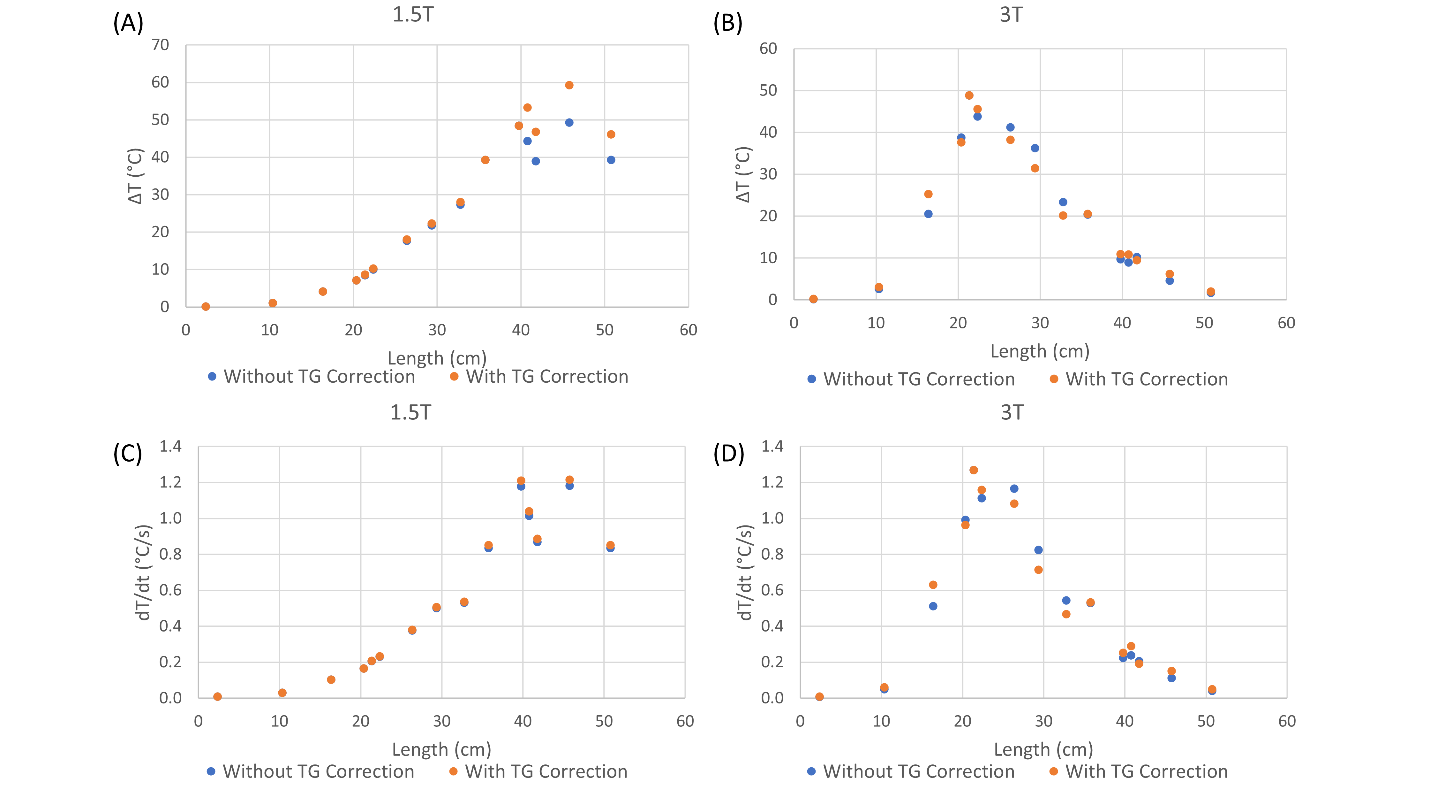


Figure S1: Overall heating can be determined from either the initial slope *dT*/*dt* or from the maximum temperature rise. (A,B) Maximal change in temperature versus wire length, with and without transmit gain correction at (A) 1.5T and (B) 3T. (C,D) Initial slope *dT/dt* plotted versus length with and without transmit gain correction at (C) 1.5T and (D) 3T. Both methods provided similar results, and the maximum temperature rise (A) and (B) was used in subsequent analysis.
