# Supplementary material for: Modeling and measurement of lead tip heating and resonant length for implanted, insulated wires
Source: Magn Reson Med. Author manuscript; Available in PMC 2025 Oct 1. (PMC11414523; doi:10.1002/mrm.30145)
Supplement: Tab S1 [file NIHMS1990163-supplement-Tab_S1.docx]

Table S1: Transmit Gain Corrections

| **3T** | | | | |
| --- | --- | --- | --- | --- |
| **Length (cm)** | **Measured ΔT (°C)** | **Average APS** | **Factor to Normalize SAR** | **Corrected Temperature Rise (°C)** |
| 2.38 | 0.14 | 88.25 | 1.34 | 0.19 |
| 10.38 | 2.55 | 83.50 | 1.20 | 3.07 |
| 16.38 | 20.50 | 84.50 | 1.23 | 25.22 |
| 20.38 | 38.74 | 74.25 | 0.97 | 37.64 |
| 21.38 | 48.86 | 75.50 | 1.00 | 48.86 |
| 22.38 | 43.78 | 77.25 | 1.04 | 45.58 |
| 26.38 | 41.22 | 72.25 | 0.93 | 38.25 |
| 29.38 | 36.25 | 69.25 | 0.87 | 31.39 |
| 32.78 | 23.35 | 69.00 | 0.86 | 20.10 |
| 35.78 | 20.39 | 75.75 | 1.01 | 20.51 |
| 39.78 | 9.65 | 80.75 | 1.13 | 10.89 |
| 40.78 | 8.91 | 84.00 | 1.22 | 10.84 |
| 41.78 | 10.19 | 72.25 | 0.93 | 9.46 |
| 45.78 | 4.56 | 88.75 | 1.36 | 6.19 |
| 50.78 | 1.63 | 83.50 | 1.20 | 1.96 |
| **Scanner Predicted SAR (W/kg):** 1.00 | | | | |
| **Effective SAR (W/kg):** 2.63 | | | | |
| **1.5T** | | | | |
| **Length (cm)** | **Measured ΔT (°C)** | **Average APS** | **Factor to Normalize SAR** | **Corrected Temperature Rise (°C)** |
| 2.38 | 0.13 | 142.00 | 1.00 | 0.13 |
| 10.38 | 1.06 | 142.00 | 1.00 | 1.06 |
| 16.38 | 4.11 | 142.00 | 1.00 | 4.11 |
| 20.38 | 7.09 | 142.00 | 1.00 | 7.09 |
| 21.38 | 8.45 | 143.00 | 1.02 | 8.65 |
| 22.38 | 9.98 | 143.00 | 1.02 | 10.21 |
| 26.38 | 17.63 | 143.00 | 1.02 | 18.04 |
| 29.38 | 21.73 | 143.00 | 1.02 | 22.24 |
| 32.78 | 27.32 | 143.00 | 1.02 | 27.96 |
| 35.78 | 39.23 | 142.00 | 1.00 | 39.23 |
| 39.78 | 48.37 | 142.00 | 1.00 | 48.37 |
| 40.78 | 44.32 | 150.00 | 1.20 | 53.28 |
| 41.78 | 38.93 | 150.00 | 1.20 | 46.80 |
| 45.78 | 49.29 | 150.00 | 1.20 | 59.26 |
| 50.78 | 39.23 | 149.00 | 1.17 | 46.09 |
| **Scanner Predicted SAR (W/kg):** 2.14 | | | | |
| **Effective SAR (W/kg):** 0.725 | | | | |

Table S1: This table lists the measured temperature rise, average autoprescan values, and the transmit gain factor that was used to calculate the corrected temperature rise. The table also lists the FSE sequence predicted whole-body SAR that was calculated by scanner software, as well as the effective SAR considering the transmit gain factor. The scanner predicted SAR and effective predicted SAR differ substantially, because for the latter the transmit gain (TG_s_, see main text) applied at each field strength was selected so that the observed temperature rise at the resonant length was approximately 50^o^C. The factor to normalize SAR is closer to 1, and accounts for small variation in coil loading affected transmit gain among the various lengths of wires. For this factor, the reference wire for 3T was 21.38cm and the reference wire for 1.5T was 2.38cm APS: autoprescan, SAR: specific absorption rate, FSE: fast spin echo
