# Supplementary material for: Modeling and measurement of lead tip heating and resonant length for implanted, insulated wires
Source: Magn Reson Med. Author manuscript; Available in PMC 2025 Oct 1. (PMC11414523; doi:10.1002/mrm.30145)
Supplement: Fig S2 [file NIHMS1990163-supplement-Fig_S2.docx]

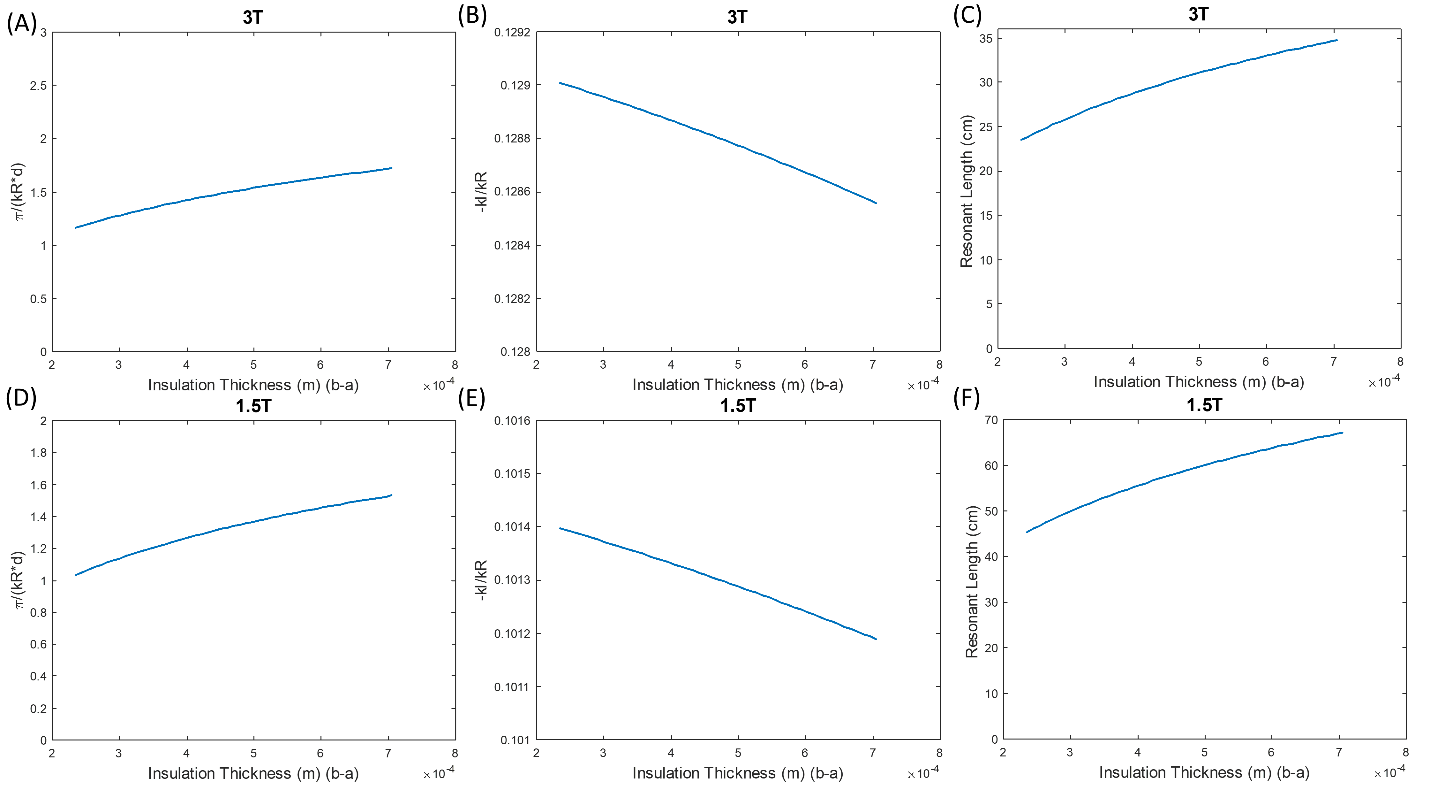


Figure S2: Predicted resonant length using the transmission line model (TLM) with an optimized value of $Г$ for a simulated electric field at 1.5T (A-C) and 3T (D-F). (A&D) show $\pi/(k_{R}d)$versus insulation thickness, with *d* = 45 and 21 cm at 1.5T and 3T, respectively. (B&E) plot $-k_{I}/k_{R}$ versus insulation thickness. (C&F) plot resonant length versus insulation thickness.
